# 16-*O*-Methyl­cafestol

**DOI:** 10.1107/S1600536810007920

**Published:** 2010-03-06

**Authors:** Xia-Li Liao, Xiao-Zhen Chen, Kai-Bei Yu, Guo-You Li

**Affiliations:** a Chengdu Institute of Biology, Chinese Academy of Sciences, Chengdu 610041, People’s Republic of China; b Chengdu Institute of Organic Chemistry, Chinese Academy of Sciences, Chengdu 610041, People’s Republic of China

## Abstract

The title compound [systematic name: (3b*S*,5a*S*,7*R*,8*R*,10a*R*,10b*S*)-7-meth­oxy-10b-methyl-3b,4,5,6,7,8,9,10,10a,10b,11,12-dodeca­hydro-5a,8-methano-5a*H*-cyclo­hepta­l[5,6]naph­tho[2,1-*b*]furan-7-methanol], C_21_H_30_O_3_, was isolated from the beans of *Coffea robusta*. The mol­ecule contains five fused rings including a furan ring. The two six-membered rings are in chair conformations, but the third six-membered ring and the five-membered aliphatic ring adopt envelope conformations. Inter­molecular O—H⋯O hydrogen bonding is present in the crystal structure.

## Related literature

For related structures, see: Beattie & Mills (1955 ▶); Djerassi *et al.* (1959 ▶); Finnegan & Djerassi (1960 ▶); Scott *et al.* (1962 ▶); Ducruix *et al.* (1977 ▶); Chakrabarti & Venkatesan (1981 ▶). For a total synthesis of cafestol, see: Corey *et al.* (1987 ▶). For the absolute configuration of a related compound, see: Djerassi *et al.* (1953 ▶). For the relative configuration, see: Scharnhop & Winterhalter (2009 ▶).
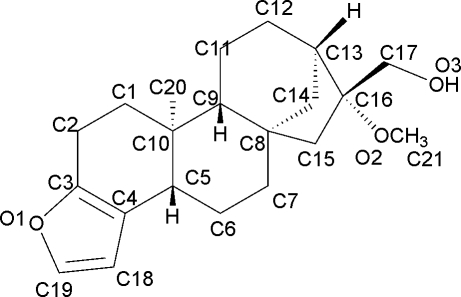


## Experimental

### 

#### Crystal data

C_21_H_30_O_3_
*M* *_r_* = 330.45
Monoclinic, 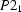

*a* = 10.6399 (9) Å
*b* = 7.0001 (5) Å
*c* = 11.5765 (12) Å
β = 92.640 (5)°
*V* = 861.31 (13) Å^3^
*Z* = 2
Mo *K*α radiation
μ = 0.08 mm^−1^
*T* = 93 K
0.50 × 0.33 × 0.20 mm

#### Data collection

Rigaku SPIDER diffractometer
6921 measured reflections
2116 independent reflections
1961 reflections with *I* > 2σ(*I*)
*R*_int_ = 0.029

#### Refinement

*R*[*F* ^2^ > 2σ(*F* ^2^)] = 0.031
*wR*(*F* ^2^) = 0.069
*S* = 1.00
2116 reflections
223 parameters
1 restraint
H atoms treated by a mixture of independent and constrained refinement
Δρ_max_ = 0.22 e Å^−3^
Δρ_min_ = −0.15 e Å^−3^

### 

Data collection: *RAPID-AUTO* (Rigaku, 2004 ▶); cell refinement: *RAPID-AUTO*; data reduction: *RAPID-AUTO*; program(s) used to solve structure: *SHELXS97* (Sheldrick, 2008 ▶); program(s) used to refine structure: *SHELXL97* (Sheldrick, 2008 ▶); molecular graphics: *ORTEP-3 for Windows* (Farrugia, 1997 ▶); software used to prepare material for publication: *SHELXL97*.

## Supplementary Material

Crystal structure: contains datablocks global, I. DOI: 10.1107/S1600536810007920/xu2727sup1.cif

Structure factors: contains datablocks I. DOI: 10.1107/S1600536810007920/xu2727Isup2.hkl

Additional supplementary materials:  crystallographic information; 3D view; checkCIF report

## Figures and Tables

**Table 1:** Hydrogen-bond geometry (Å, °)

*D*—H⋯*A*	*D*—H	H⋯*A*	*D*⋯*A*	*D*—H⋯*A*
O3—H3*O*⋯O2^i^	0.81 (3)	1.97 (3)	2.7479 (19)	163 (3)

Symmetry code: (i) 
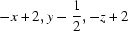
.

**Fig. 1.**
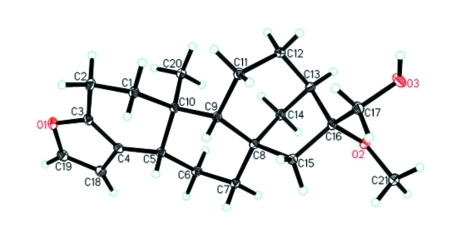
View of the title molecule showing the atom-numbering scheme. Displacement ellipsoids are drawn at the 50% probability level.

**Fig. 2.**
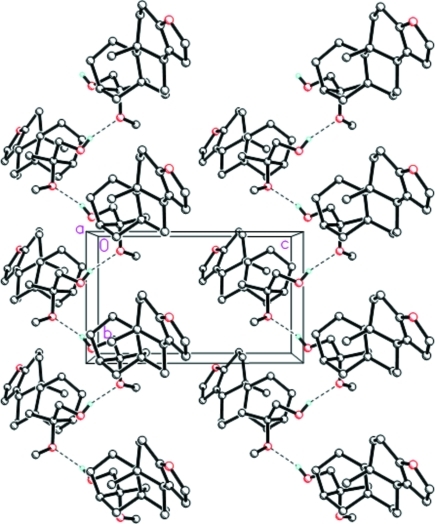
The crystal packing of the title molecule, viewed down the a axis. H atoms were omitted for clarity.

## supplementary crystallographic information

### Comment

*Coffea robusta* is a species of coffee which has its origins in western Africa. As a part of our research on the bioactive constituents in coffee, the title compound was isolated. Its relative configuration was obtained from ESI-MS and NMR analyses, which were compared with a recent report (Scharnhop *et al.*, 2009), and confirmed by Single-crystal X-ray diffraction study. The molecule of the title compound contains a five-ring system A/B/C/D/E(Fig. 1). There is a *trans* junction between ring A(C1—C5/C10)and ring B(C5—C10). *Cis* junction are present between ring B and ring C(C8—C9/C11—C14) and ring C and ring D(C8/C13—C16). Ring A and D are both in envelope-like conformations, with C10 and C16 at the flap, respectively. Ring B and C both adopt chair conformations. The furan ring E(C5—C6/C18—C19/O1), of course, is planar. Intermolecular O—H···O hydrogen bonding helps to stabilize the crystal structure(Fig. 2).

### Experimental

The powdered seeds of *Coffea robusta* were extracted with cyclohexane and filtered. The filtrate was evaporated under reduced pressure. Then the residue was hydrolyzed with KOH in EtOH and extracted with *tert*-Butyl methyl ether(TBME). The extract was chromatograhed over Silica gel column with eluent of petroleum ether/ethyl acetate(3:1) to provide the title compound as white solid. It was recrystallized in acetone to afford suitable crystals for Single-crystal X-ray diffraction analysis.

### Refinement

Hydroxyl H atom was located in a difference Fourier map and was refined isotropically. Other H atoms were located geometrically with C—H = 0.95-1.00 Å, and were refined in riding mode with U_iso_(H) = 1.2U_eq_(C). The absolute configuration could not be determined from the X-ray analysis, owing to the absence of significant anomalous scattering, and Friedel pairs were merged. The absolute configuration was assigned by a comparison between the measured Optical Rotatory Power ([α]^24^_D_ = -121° (c=0.4, CHCl_3_)) and a previous work (For Cafestol: [α]^24^_D_ = -97° (CHCl_3_)) (Djerassi *et al.*, 1953).

### Crystal data

**Table:**

C_21_H_30_O_3_	*F*(000) = 360
*M**_r_* = 330.45	*D*_x_ = 1.274 Mg m^−^^3^
Monoclinic, *P*2_1_	Melting point: 448 K
Hall symbol: P 2yb	Mo *K*α radiation, λ = 0.71073 Å
*a* = 10.6399 (9) Å	Cell parameters from 2892 reflections
*b* = 7.0001 (5) Å	θ = 3.4–27.5°
*c* = 11.5765 (12) Å	µ = 0.08 mm^−^^1^
β = 92.640 (5)°	*T* = 93 K
*V* = 861.31 (13) Å^3^	Prism, colorless
*Z* = 2	0.50 × 0.33 × 0.20 mm

### Data collection

**Table:**

Rigaku SPIDER diffractometer	1961 reflections with *I* > 2σ(*I*)
Radiation source: Rotating Anode	*R*_int_ = 0.029
graphite	θ_max_ = 27.5°, θ_min_ = 3.4°
ω scans	*h* = −12→13
6921 measured reflections	*k* = −9→9
2116 independent reflections	*l* = −15→13

### Refinement

**Table:**

Refinement on *F*^2^	Primary atom site location: structure-invariant direct methods
Least-squares matrix: full	Secondary atom site location: difference Fourier map
*R*[*F*^2^ > 2σ(*F*^2^)] = 0.031	Hydrogen site location: inferred from neighbouring sites
*wR*(*F*^2^) = 0.069	H atoms treated by a mixture of independent and constrained refinement
*S* = 1.00	*w* = 1/[σ^2^(*F*_o_^2^) + (0.0309*P*)^2^ + 0.16*P*] where *P* = (*F*_o_^2^ + 2*F*_c_^2^)/3
2116 reflections	(Δ/σ)_max_ < 0.001
223 parameters	Δρ_max_ = 0.22 e Å^−^^3^
1 restraint	Δρ_min_ = −0.15 e Å^−^^3^

### Special details

**Table:**

Experimental. ^13^C NMR (150 MHz, CDCl_3_, δ, p.p.m.): 148.8(C3), 140.6(C19), 120.1(C4), 108.3(C18), 87.0(C16), 60.5(C17), 52.1(C5), 49.1(C15), 48.9(C21), 44.4(C8), 44.3(C9), 41.5(C13), 41.0(C7), 38.7(C10), 37.8(C14), 35.8(C1), 25.7(C12), 23.1(C6), 20.6(C2), 19.2(C11), 13.3(C20).
Geometry. All esds (except the esd in the dihedral angle between two l.s. planes) are estimated using the full covariance matrix. The cell esds are taken into account individually in the estimation of esds in distances, angles and torsion angles; correlations between esds in cell parameters are only used when they are defined by crystal symmetry. An approximate (isotropic) treatment of cell esds is used for estimating esds involving l.s. planes.
Refinement. Refinement of *F*^2^ against ALL reflections. The weighted *R*-factor wR and goodness of fit *S* are based on *F*^2^, conventional *R*-factors *R* are based on *F*, with *F* set to zero for negative *F*^2^. The threshold expression of *F*^2^ > σ(*F*^2^) is used only for calculating *R*-factors(gt) etc. and is not relevant to the choice of reflections for refinement. *R*-factors based on *F*^2^ are statistically about twice as large as those based on *F*, and *R*- factors based on ALL data will be even larger.

### Fractional atomic coordinates and isotropic or equivalent isotropic displacement parameters (Å^2^)

**Table:**

	*x*	*y*	*z*	*U*_iso_*/*U*_eq_	
O1	0.09240 (11)	0.1142 (2)	0.60603 (10)	0.0202 (3)	
O2	0.91263 (11)	0.64850 (18)	0.83888 (10)	0.0180 (3)	
O3	1.04079 (14)	0.3897 (2)	0.97682 (12)	0.0259 (3)	
C1	0.41584 (16)	0.0015 (3)	0.73392 (16)	0.0173 (4)	
H1A	0.4554	−0.0512	0.6651	0.021*	
H1B	0.4492	−0.0705	0.8022	0.021*	
C2	0.27216 (16)	−0.0310 (3)	0.72069 (16)	0.0196 (4)	
H2A	0.2544	−0.1577	0.6857	0.024*	
H2B	0.2349	−0.0272	0.7975	0.024*	
C3	0.21656 (16)	0.1217 (3)	0.64550 (14)	0.0167 (4)	
C4	0.27270 (16)	0.2813 (3)	0.60818 (14)	0.0159 (4)	
C5	0.41066 (16)	0.3158 (3)	0.63354 (15)	0.0148 (4)	
H5	0.4551	0.2495	0.5707	0.018*	
C6	0.45291 (16)	0.5236 (3)	0.62997 (15)	0.0174 (4)	
H6A	0.4238	0.5930	0.6983	0.021*	
H6B	0.4167	0.5865	0.5594	0.021*	
C7	0.59643 (16)	0.5269 (3)	0.62984 (15)	0.0175 (4)	
H7A	0.6254	0.6613	0.6279	0.021*	
H7B	0.6238	0.4632	0.5589	0.021*	
C8	0.65845 (16)	0.4275 (3)	0.73580 (14)	0.0148 (4)	
C9	0.60201 (16)	0.2259 (2)	0.75520 (15)	0.0141 (4)	
H9	0.6291	0.1485	0.6881	0.017*	
C10	0.45483 (16)	0.2142 (3)	0.74799 (15)	0.0145 (4)	
C11	0.66860 (16)	0.1311 (3)	0.86261 (14)	0.0175 (4)	
H11A	0.6107	0.0350	0.8935	0.021*	
H11B	0.7433	0.0617	0.8367	0.021*	
C12	0.71155 (17)	0.2649 (3)	0.96239 (15)	0.0188 (4)	
H12A	0.6400	0.2854	1.0127	0.023*	
H12B	0.7793	0.2009	1.0094	0.023*	
C13	0.75979 (16)	0.4604 (3)	0.92313 (15)	0.0161 (4)	
H13	0.7822	0.5436	0.9912	0.019*	
C14	0.65661 (17)	0.5522 (3)	0.84587 (15)	0.0167 (4)	
H14A	0.6765	0.6872	0.8288	0.020*	
H14B	0.5740	0.5457	0.8817	0.020*	
C15	0.80304 (16)	0.4041 (3)	0.72158 (14)	0.0166 (4)	
H15A	0.8227	0.2716	0.6986	0.020*	
H15B	0.8319	0.4923	0.6614	0.020*	
C16	0.86878 (16)	0.4516 (3)	0.84003 (15)	0.0159 (4)	
C17	0.97698 (17)	0.3184 (3)	0.87504 (15)	0.0198 (4)	
H17A	0.9441	0.1887	0.8897	0.024*	
H17B	1.0362	0.3099	0.8117	0.024*	
C18	0.17903 (17)	0.3819 (3)	0.53822 (16)	0.0201 (4)	
H18	0.1897	0.4990	0.4983	0.024*	
C19	0.07310 (17)	0.2766 (3)	0.54064 (16)	0.0213 (4)	
H19	−0.0046	0.3101	0.5023	0.026*	
C20	0.39111 (16)	0.2962 (3)	0.85393 (15)	0.0181 (4)	
H20A	0.4278	0.2371	0.9244	0.022*	
H20B	0.3007	0.2690	0.8475	0.022*	
H20C	0.4043	0.4347	0.8573	0.022*	
C21	1.01924 (17)	0.6855 (3)	0.77196 (17)	0.0233 (4)	
H21A	1.0082	0.6217	0.6968	0.028*	
H21B	1.0953	0.6368	0.8129	0.028*	
H21C	1.0275	0.8235	0.7601	0.028*	
H3O	1.052 (2)	0.302 (4)	1.021 (2)	0.043 (8)*	

### Atomic displacement parameters (Å^2^)

**Table:**

	*U*^11^	*U*^22^	*U*^33^	*U*^12^	*U*^13^	*U*^23^
O1	0.0154 (6)	0.0215 (7)	0.0235 (7)	−0.0012 (6)	−0.0020 (5)	−0.0019 (6)
O2	0.0194 (6)	0.0162 (7)	0.0184 (6)	−0.0047 (5)	0.0000 (5)	0.0010 (5)
O3	0.0326 (8)	0.0219 (8)	0.0221 (7)	−0.0020 (6)	−0.0116 (6)	0.0034 (6)
C1	0.0173 (9)	0.0132 (9)	0.0210 (9)	−0.0003 (7)	−0.0024 (7)	0.0013 (7)
C2	0.0195 (9)	0.0148 (9)	0.0245 (10)	−0.0037 (7)	−0.0004 (7)	0.0005 (7)
C3	0.0129 (8)	0.0196 (9)	0.0172 (8)	−0.0006 (8)	−0.0015 (6)	−0.0029 (8)
C4	0.0178 (9)	0.0166 (9)	0.0135 (8)	0.0014 (7)	0.0009 (6)	−0.0031 (7)
C5	0.0152 (8)	0.0135 (9)	0.0157 (8)	−0.0008 (7)	0.0002 (6)	−0.0001 (7)
C6	0.0190 (9)	0.0149 (9)	0.0181 (9)	−0.0007 (7)	−0.0007 (7)	0.0046 (7)
C7	0.0180 (9)	0.0169 (9)	0.0174 (9)	−0.0043 (7)	−0.0002 (7)	0.0034 (7)
C8	0.0156 (8)	0.0135 (8)	0.0154 (8)	−0.0015 (7)	0.0008 (6)	0.0010 (7)
C9	0.0145 (8)	0.0129 (8)	0.0148 (9)	−0.0011 (7)	0.0007 (6)	−0.0016 (7)
C10	0.0156 (8)	0.0123 (8)	0.0154 (9)	−0.0008 (7)	−0.0009 (7)	0.0012 (7)
C11	0.0181 (9)	0.0138 (8)	0.0204 (9)	−0.0018 (8)	−0.0016 (7)	0.0021 (8)
C12	0.0204 (9)	0.0207 (10)	0.0152 (9)	−0.0047 (8)	−0.0016 (7)	0.0031 (8)
C13	0.0187 (9)	0.0160 (9)	0.0135 (8)	−0.0038 (7)	0.0007 (7)	−0.0025 (7)
C14	0.0170 (9)	0.0132 (8)	0.0199 (9)	−0.0024 (7)	0.0018 (7)	−0.0026 (7)
C15	0.0164 (8)	0.0181 (9)	0.0155 (8)	−0.0021 (7)	0.0014 (6)	−0.0003 (7)
C16	0.0168 (9)	0.0133 (9)	0.0176 (9)	−0.0038 (7)	−0.0011 (7)	−0.0005 (7)
C17	0.0199 (9)	0.0204 (10)	0.0189 (9)	−0.0016 (8)	−0.0030 (7)	−0.0015 (8)
C18	0.0207 (9)	0.0206 (10)	0.0190 (9)	0.0023 (8)	−0.0006 (7)	−0.0002 (8)
C19	0.0182 (9)	0.0253 (10)	0.0201 (9)	0.0035 (8)	−0.0024 (7)	0.0001 (8)
C20	0.0167 (8)	0.0204 (9)	0.0171 (9)	−0.0037 (8)	0.0007 (7)	0.0000 (8)
C21	0.0212 (9)	0.0271 (11)	0.0215 (10)	−0.0078 (8)	0.0002 (7)	0.0039 (8)

### Geometric parameters (Å, °)

**Table:**

O1—C19	1.376 (2)	C9—C10	1.566 (2)
O1—C3	1.379 (2)	C9—H9	1.0000
O2—C21	1.427 (2)	C10—C20	1.539 (2)
O2—C16	1.455 (2)	C11—C12	1.540 (2)
O3—C17	1.423 (2)	C11—H11A	0.9900
O3—H3O	0.81 (3)	C11—H11B	0.9900
C1—C2	1.546 (2)	C12—C13	1.538 (3)
C1—C10	1.552 (2)	C12—H12A	0.9900
C1—H1A	0.9900	C12—H12B	0.9900
C1—H1B	0.9900	C13—C14	1.526 (3)
C2—C3	1.484 (3)	C13—C16	1.542 (2)
C2—H2A	0.9900	C13—H13	1.0000
C2—H2B	0.9900	C14—H14A	0.9900
C3—C4	1.347 (3)	C14—H14B	0.9900
C4—C18	1.439 (2)	C15—C16	1.547 (2)
C4—C5	1.503 (2)	C15—H15A	0.9900
C5—C6	1.524 (3)	C15—H15B	0.9900
C5—C10	1.557 (2)	C16—C17	1.522 (3)
C5—H5	1.0000	C17—H17A	0.9900
C6—C7	1.527 (2)	C17—H17B	0.9900
C6—H6A	0.9900	C18—C19	1.348 (3)
C6—H6B	0.9900	C18—H18	0.9500
C7—C8	1.533 (2)	C19—H19	0.9500
C7—H7A	0.9900	C20—H20A	0.9800
C7—H7B	0.9900	C20—H20B	0.9800
C8—C14	1.546 (2)	C20—H20C	0.9800
C8—C9	1.554 (2)	C21—H21A	0.9800
C8—C15	1.563 (2)	C21—H21B	0.9800
C9—C11	1.552 (2)	C21—H21C	0.9800
			
C19—O1—C3	105.49 (14)	C9—C11—H11A	108.1
C21—O2—C16	116.12 (14)	C12—C11—H11B	108.1
C17—O3—H3O	107.8 (19)	C9—C11—H11B	108.1
C2—C1—C10	114.20 (14)	H11A—C11—H11B	107.3
C2—C1—H1A	108.7	C13—C12—C11	114.28 (14)
C10—C1—H1A	108.7	C13—C12—H12A	108.7
C2—C1—H1B	108.7	C11—C12—H12A	108.7
C10—C1—H1B	108.7	C13—C12—H12B	108.7
H1A—C1—H1B	107.6	C11—C12—H12B	108.7
C3—C2—C1	108.51 (15)	H12A—C12—H12B	107.6
C3—C2—H2A	110.0	C14—C13—C12	107.89 (15)
C1—C2—H2A	110.0	C14—C13—C16	101.10 (14)
C3—C2—H2B	110.0	C12—C13—C16	114.82 (15)
C1—C2—H2B	110.0	C14—C13—H13	110.9
H2A—C2—H2B	108.4	C12—C13—H13	110.9
C4—C3—O1	110.95 (16)	C16—C13—H13	110.9
C4—C3—C2	127.93 (15)	C13—C14—C8	102.08 (14)
O1—C3—C2	121.11 (16)	C13—C14—H14A	111.4
C3—C4—C18	106.28 (15)	C8—C14—H14A	111.4
C3—C4—C5	120.92 (16)	C13—C14—H14B	111.4
C18—C4—C5	132.55 (17)	C8—C14—H14B	111.4
C4—C5—C6	115.76 (15)	H14A—C14—H14B	109.2
C4—C5—C10	110.29 (14)	C16—C15—C8	106.93 (13)
C6—C5—C10	112.40 (14)	C16—C15—H15A	110.3
C4—C5—H5	105.9	C8—C15—H15A	110.3
C6—C5—H5	105.9	C16—C15—H15B	110.3
C10—C5—H5	105.9	C8—C15—H15B	110.3
C5—C6—C7	108.12 (15)	H15A—C15—H15B	108.6
C5—C6—H6A	110.1	O2—C16—C17	110.11 (14)
C7—C6—H6A	110.1	O2—C16—C13	102.62 (14)
C5—C6—H6B	110.1	C17—C16—C13	116.09 (15)
C7—C6—H6B	110.1	O2—C16—C15	109.12 (14)
H6A—C6—H6B	108.4	C17—C16—C15	114.21 (15)
C6—C7—C8	112.69 (14)	C13—C16—C15	103.85 (14)
C6—C7—H7A	109.1	O3—C17—C16	109.38 (16)
C8—C7—H7A	109.1	O3—C17—H17A	109.8
C6—C7—H7B	109.1	C16—C17—H17A	109.8
C8—C7—H7B	109.1	O3—C17—H17B	109.8
H7A—C7—H7B	107.8	C16—C17—H17B	109.8
C7—C8—C14	112.44 (15)	H17A—C17—H17B	108.2
C7—C8—C9	111.91 (14)	C19—C18—C4	106.20 (17)
C14—C8—C9	111.97 (14)	C19—C18—H18	126.9
C7—C8—C15	110.65 (13)	C4—C18—H18	126.9
C14—C8—C15	101.29 (13)	C18—C19—O1	111.06 (16)
C9—C8—C15	108.00 (14)	C18—C19—H19	124.5
C11—C9—C8	109.82 (14)	O1—C19—H19	124.5
C11—C9—C10	116.03 (14)	C10—C20—H20A	109.5
C8—C9—C10	115.59 (14)	C10—C20—H20B	109.5
C11—C9—H9	104.7	H20A—C20—H20B	109.5
C8—C9—H9	104.7	C10—C20—H20C	109.5
C10—C9—H9	104.7	H20A—C20—H20C	109.5
C20—C10—C1	108.40 (15)	H20B—C20—H20C	109.5
C20—C10—C5	112.40 (14)	O2—C21—H21A	109.5
C1—C10—C5	106.25 (14)	O2—C21—H21B	109.5
C20—C10—C9	114.45 (14)	H21A—C21—H21B	109.5
C1—C10—C9	108.50 (14)	O2—C21—H21C	109.5
C5—C10—C9	106.46 (14)	H21A—C21—H21C	109.5
C12—C11—C9	116.73 (16)	H21B—C21—H21C	109.5
C12—C11—H11A	108.1		
			
C10—C1—C2—C3	40.0 (2)	C11—C9—C10—C1	−65.34 (19)
C19—O1—C3—C4	0.62 (19)	C8—C9—C10—C1	163.96 (14)
C19—O1—C3—C2	179.58 (16)	C11—C9—C10—C5	−179.33 (14)
C1—C2—C3—C4	−10.1 (3)	C8—C9—C10—C5	49.96 (19)
C1—C2—C3—O1	171.15 (15)	C8—C9—C11—C12	33.6 (2)
O1—C3—C4—C18	−1.1 (2)	C10—C9—C11—C12	−99.74 (18)
C2—C3—C4—C18	−179.94 (18)	C9—C11—C12—C13	−36.3 (2)
O1—C3—C4—C5	−176.03 (15)	C11—C12—C13—C14	55.56 (19)
C2—C3—C4—C5	5.1 (3)	C11—C12—C13—C16	−56.3 (2)
C3—C4—C5—C6	−157.29 (16)	C12—C13—C14—C8	−70.78 (16)
C18—C4—C5—C6	29.3 (3)	C16—C13—C14—C8	50.07 (16)
C3—C4—C5—C10	−28.3 (2)	C7—C8—C14—C13	−160.51 (14)
C18—C4—C5—C10	158.29 (18)	C9—C8—C14—C13	72.48 (16)
C4—C5—C6—C7	−167.16 (13)	C15—C8—C14—C13	−42.38 (16)
C10—C5—C6—C7	64.86 (18)	C7—C8—C15—C16	138.40 (15)
C5—C6—C7—C8	−58.46 (19)	C14—C8—C15—C16	18.98 (18)
C6—C7—C8—C14	−77.13 (19)	C9—C8—C15—C16	−98.79 (16)
C6—C7—C8—C9	49.9 (2)	C21—O2—C16—C17	53.11 (19)
C6—C7—C8—C15	170.41 (15)	C21—O2—C16—C13	177.30 (14)
C7—C8—C9—C11	179.55 (14)	C21—O2—C16—C15	−72.99 (18)
C14—C8—C9—C11	−53.16 (18)	C14—C13—C16—O2	76.47 (16)
C15—C8—C9—C11	57.52 (17)	C12—C13—C16—O2	−167.70 (14)
C7—C8—C9—C10	−46.8 (2)	C14—C13—C16—C17	−163.39 (15)
C14—C8—C9—C10	80.45 (18)	C12—C13—C16—C17	−47.6 (2)
C15—C8—C9—C10	−168.87 (14)	C14—C13—C16—C15	−37.17 (17)
C2—C1—C10—C20	57.47 (19)	C12—C13—C16—C15	78.65 (18)
C2—C1—C10—C5	−63.55 (19)	C8—C15—C16—O2	−97.88 (16)
C2—C1—C10—C9	−177.68 (14)	C8—C15—C16—C17	138.41 (16)
C4—C5—C10—C20	−64.11 (19)	C8—C15—C16—C13	11.01 (19)
C6—C5—C10—C20	66.70 (18)	O2—C16—C17—O3	49.13 (19)
C4—C5—C10—C1	54.31 (18)	C13—C16—C17—O3	−66.9 (2)
C6—C5—C10—C1	−174.88 (14)	C15—C16—C17—O3	172.30 (15)
C4—C5—C10—C9	169.83 (14)	C3—C4—C18—C19	1.1 (2)
C6—C5—C10—C9	−59.36 (18)	C5—C4—C18—C19	175.23 (18)
C11—C9—C10—C20	55.9 (2)	C4—C18—C19—O1	−0.8 (2)
C8—C9—C10—C20	−74.85 (19)	C3—O1—C19—C18	0.1 (2)

### Hydrogen-bond geometry (Å, °)

**Table:**

*D*—H···*A*	*D*—H	H···*A*	*D*···*A*	*D*—H···*A*
O3—H3O···O2^i^	0.81 (3)	1.97 (3)	2.7479 (19)	163 (3)

Symmetry codes: (i) −*x*+2, *y*−1/2, −*z*+2.
